# Regulation of insulin secretion in mouse islets: metabolic amplification by alpha-ketoisocaproate coincides with rapid and sustained increase in acetyl-CoA content

**DOI:** 10.1007/s00210-022-02290-8

**Published:** 2022-11-10

**Authors:** Uwe Panten, Dennis Brüning, Ingo Rustenbeck

**Affiliations:** Institute of Pharmacology, Toxicology and Clinical Pharmacy, Technische Universität Braunschweig, Mendelssohnstr. 1, 38106 Braunschweig, Germany

**Keywords:** Acetyl-CoA, Alpha-ketoisocaproate, Beta-cell, Glucose, Insulin secretion, Metabolic amplification

## Abstract

Glucose and alpha-ketoisocaproate, the keto acid analogue of leucine, stimulate insulin secretion in the absence of other exogenous fuels. Their mitochondrial metabolism in the beta-cell raises the cytosolic ATP/ADP ratio, thereby providing the triggering signal for the exocytosis of the insulin granules. However, additional amplifying signals are required for the full extent of insulin secretion stimulated by these fuels. While it is generally recognized that the amplifying signals are also derived from the mitochondrial metabolism, their exact nature is still unclear. The current study tests the hypothesis that the supply of cytosolic acetyl-CoA is a signal in the amplifying pathway. The contents of acetyl-CoA and acetyl-CoA plus CoA-SH were measured in isolated mouse islets. Insulin secretion was recorded in isolated perifused islets. In islets, the ATP-sensitive K^+^ channels of which were pharmacologically closed and which were preincubated without exogenous fuel, 10 mmol/L alpha-ketoisocaproate enhanced the acetyl-CoA content after 5 and 20 min incubations and decreased the acetyl-CoA plus CoA-SH within 5 min, but not after 20 min. In islets not exposed to drugs, the preincubation with 3 mmol/L glucose, a non-triggering concentration, elevated the acetyl-CoA content. This content was further increased after 5 min and 20 min incubations with 30 mmol/L glucose, concurrent with a strong increase in insulin secretion. Alpha-ketoisocaproate and glucose increase the supply of acetyl-CoA in the beta-cell cytosol during both phases of insulin secretion. Most likely, this increase provides a signal for the metabolic amplification.

## Introduction

The regulation of insulin secretion is unique in that it requires the metabolic breakdown of the main physiological stimulus, glucose, by the pancreatic beta-cell. The events which link the metabolism to the electrical activity of the beta-cell and to depolarization-induced Ca^2+^ influx are named “triggering pathway” (Henquin 2000). In addition, the beta-cell metabolism intensifies the efficiency of elevated cytosolic Ca^2+^. This hitherto incompletely understood process is named “amplifying pathway” (Henquin 2000) and is induced by the mitochondrial export of metabolites (Jitrapakdee et al. 2010; Prentki et al. 2013).

**Fig. 1:**
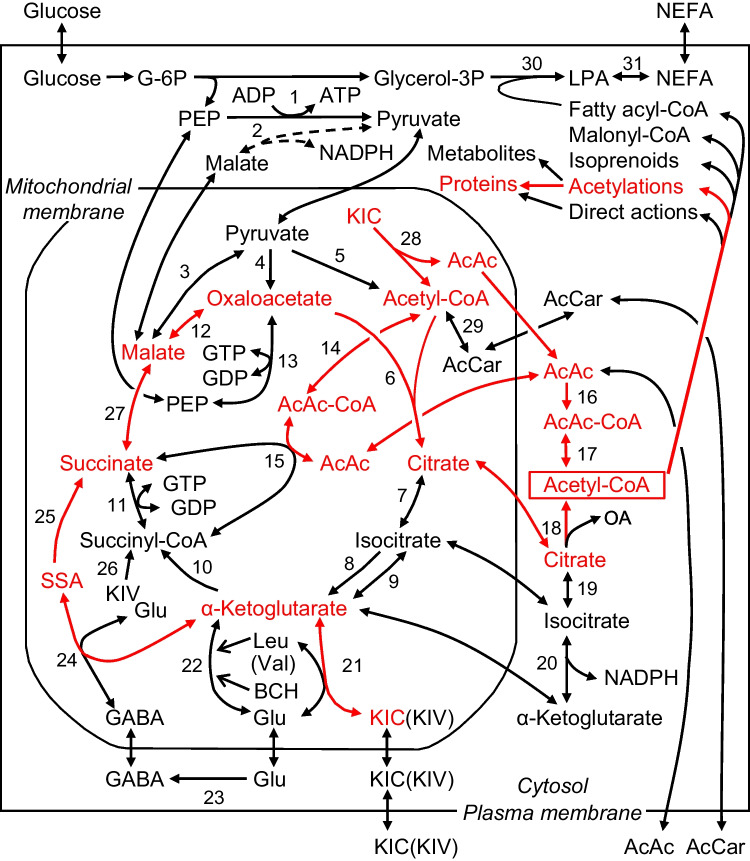
Fuel-induced supply and acute effects of cytosolic acetyl-CoA in beta-cells. Direct actions mean actions without molecular transformation of acetyl-CoA. The pathway probably mediating alpha-ketoisocaproate-induced generation of cytosolic acetyl-CoA and amplification is indicated in red. For clarity, not all compounds participating in enzyme reactions and transport processes are shown. In the mitochondrium, formation of NADH, NADPH, and FADH_2_ and the shuttling of reducing equivalents and the transaminations of alpha-ketoglutarate with apartate and alanine are not shown. The lack of cytosolic malic enzyme 1 in mouse beta cells is indicated by broken lines. Leu and BCH, but not Val, allosterically activate the glutamate dehydrogenase. GABA-shunt comprises the enzymes 23, 24, and 25. For further explanations and references, see the text. Abbreviations: Acetoacetate (AcAc), acetoacetyl-CoA (AcAc-CoA), acetyl-L-carnitine (AcCar), 2-aminobicyclo[2,2,1]heptane-2-carboxylic acid (BCH, non-metabolizable Leu analogue), glucose 6-phosphate (G-6P), glycerol 3-phosphate (glycerol-3P), alpha-ketoisocaproate (KIC), alpha-ketoisovalerate (KIV), lysophosphatidic acid (LPA), non-esterified fatty acid (NEFA), oxaloacetate (OA), phosphoenolpyruvate (PEP), succinate semialdehyde (SSA). Parentheses indicate that KIV and Val are alternative substrates and products. Numbers indicate enzymes: 1, pyruvate kinase; 2, cytosolic malic enzyme 1; 3, mitochondrial malic enzyme 2 and 3; 4, pyruvate carboxylase; 5, pyruvate dehydrogenase; 6, citrate synthase; 7, mitochondrial aconitase; 8, NAD^+^-dependent isocitrate dehydrogenase 3; 9, NADP^+^-dependent isocitrate dehydrogenase 2; 10, alpha-ketoglutarate dehydrogenase; 11, GTP-specific succinyl-CoA synthase; 12, malate dehydrogenase; 13, PEP carboxykinase; 14, acetyl-CoA transferases; 15, succinyl-CoA:3-ketoacid CoA transferase; 16, acetoacetyl-CoA synthetase; 17, acetyl-CoA transferase or acyl-CoA transferase; 18, ATP-dependent citrate lyase; 19, cytosolic aconitase; 20, cytosolic isocitrate dehydrogenase 1; 21, mitochondrial branched-chain aminotransferase; 22, glutamate dehydrogenase; 23, glutamate decarboxylase (at cytosolic faces of vesicles); 24, GABA aminotransferase; 25, succinate semialdehyde dehydrogenase; 26, branched-chain alpha-keto acid dehydrogenase and eight subsequent enzymes; 27, succinate dehydrogenase and mitochondrial fumarase; 28, branched-chain alpha-keto acid dehydrogenase and four subsequent enzymes; 29, carnitine acetyltransferase; 30, glycerol-3-phosphate acyltransferases; 31, enzymes of the glycerolipid/NEFA cycle

Triggering initiates at the glucose phosphorylation by the beta-cell glucokinase (high K_m_ hexokinase), the regulation of which plays a central role in glucose-induced insulin secretion (Lenzen 2014). The resulting increase in the glycolytic pyruvate production enhances the supply of acetyl-CoA by pyruvate dehydrogenase and of oxaloacetate by pyruvate carboxylase (Jitrapakdee et al. 2010, Prentki et al. 2013, see also Fig. 1). The provision of reducing equivalents by the citrate cycle activates the respiratory chain and ultimately elevates the cytosolic ATP/ADP ratio (Jitrapakdee et al. 2010; Prentki et al. 2013). In response to elevation of this ratio, the ATP-sensitive K^+^ channels in the beta-cell plasma membrane are closed and, in conjunction with inward currents, depolarize the beta-cell. The ensuing opening of Ca^2+^ channels raises the cytosolic free Ca^2+^ concentration and triggers insulin release (Rorsman and Ashcroft 2018).

Beta-cell mitochondria have a high activity of pyruvate carboxylase, which is unusual for non-gluconeogenic cells (MacDonald et al. 2005). The generation of oxaloacetate by pyruvate carboxylase ensures adequate supply of citrate cycle intermediates for rapid increase in citrate cycle activity and export of intermediates into the cytosol (cataplerosis) (Jitrapakdee et al. 2010; Prentki et al. 2013). The export generates signals, which amplify the insulin-releasing efficiency of the elevated cytosolic Ca^2+^ concentration (Henquin 2000). This metabolic amplification starts within a few min of fuel application and persists during the first- and second-phase secretion (Henquin 2009). Multiple metabolic intermediates in the beta-cell cytosol have been considered as putative mediators of glucose-induced amplification and objections have been raised to most of them (Jitrapakdee et al. 2010; Jensen et al. 2008; Rustenbeck et al. 2021).

In addition to glucose, only few metabolic fuels (calorigenic nutrients) by themselves trigger insulin release (MacDonald et al. 2005). Alpha-ketoisocaproate (KIC), the transamination product of leucine, has gained interest because its metabolism does not involve glycolysis and its insulin-releasing potency is about as high as the potency of glucose (Lenzen and Panten 1980; Hutton et al. 1980; Panten et al. 1981). That is why KIC reveals which glucose metabolites enhance insulin release via supportive or permissive pathways, but do not mediate amplification. In the absence of other exogenous fuels, KIC triggers insulin release at extracellular concentrations > 2–3 mmol/L and is maximally effective at 10–20 mmol/L (Lenzen and Panten 1980; Hutton et al. 1980; Panten et al. 1981). KIC triggers insulin release by serving as substrate of mitochondrial branched-chain aminotransferase in the beta-cells and thus generating alpha-ketoglutarate (Zhou et al. 2010). The transamination is maintained by the mitochondrial leucine-efflux, which is driven by the rapid leucine transport out of beta-cells (Hernández-Fisac et al. 2006), as long as mitochondrial glutamate is available. Since KIC and its catabolites were found to inhibit the alpha-ketoglutarate dehydrogenase (Pizarro-Delgado et al. 2009) and the pyruvate dehydrogenase in hepatocytes (Walajtjs-Rode and Williamson, 1980), the GABA shunt and the oxidation of KIC (Lenzen and Panten 1980; Hutton et al. 1980; Pizarro-Delgado et al. 2009) supply most of the oxaloacetate and acetyl-CoA required for activating the citrate cycle (see Fig. 1). This leads to the same events as outlined above, elevation of the cytosolic ATP/ADP ratio and closure of the ATP-sensitive K^+^ channels. In addition, KIC (> 5 mmol/L) intensifies the closure of ATP-sensitive K^+^ channels via its binding to the sulfonylurea receptor site (Heissig et al. 2005).

**Fig. 2:**
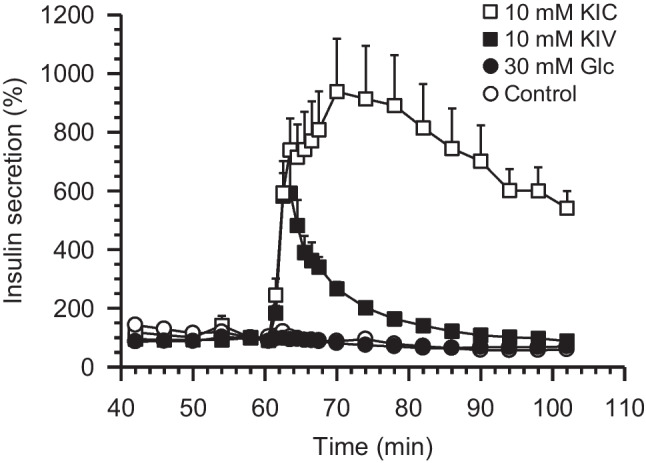
Effects of α-ketoisocaproate (KIC), α-ketoisovalerate (KIV), and glucose (Glc) on the kinetics of amplification of insulin secretion by mouse pancreatic islets. Islets were perifused from time 0 to 60 min (control period) with medium containing no substrate and from time 61 to 104 (test period) with medium containing 10 mmol/L KIC (10 KIC, white souares), 10 mmol/L KIV (10 KIV, black squares), 30 mmol/L Glc (30 Glc, black circles), or no substrate (control, white circles). All media contained 2.7 µmol/L glipizide during the control and test periods. The rate of insulín secretion is expressed as percentage of the rate at the end of the control period. Data points are the mean of 5–7 separate experiments (with SEM shown when larger than symbols) and are plotted in the middle of the sampling intervals. The figure is a modified version of Fig. 2 in Panten et al. (2013)

In a series of preceding investigations, we have examined the mechanisms of the metabolic amplification during stimulation by glucose and KIC (Urban and Panten 2005; Panten and Rustenbeck 2008; Panten et al. 2013, 2016; Schulze et al. 2017). To selectively influence the amplification, all ATP-sensitive K^+^ channels were closed by a maximally effective sulfonylurea concentration. Amplification is often examined during potassium depolarization when all ATP-sensitive K channels are opened by diazoxide (Henquin 2000), but to avoid complications resulting from interaction of diazoxide and KIC at the ATP-sensitive K^+^ channels, we prefer the depolarization by sulfonylureas. We chose mouse islets since they lack the cytosolic malic enzyme activity (MacDonald 2002), thus narrowing down the metabolites which might mediate the amplification. In islets exposed to the sulfonylurea glipizide at a maximally effective concentration throughout the experiment and pretreated by the prolonged absence of exogenous fuel, 10 mmol/L KIC within a few minutes strongly amplified the secretion of insulin, whereas the metabolic amplification by glucose stimulation was prevented (Urban and Panten 2005, Panten et al. 2013, see also Fig. 2).

Activation of the glycerolipid/non-esterified fatty acid (NEFA) cycle, which requires glycerol 3-phosphate production, and generation of NADPH by increase in supply of cytosolic isocitrate have been suggested to act as key amplifying mechanisms (Prentki et al. 2020; Campbell and Newgard 2021). But the strong amplification by 10 mmol/L KIC in mouse islets, all ATP-sensitive K^+^ channels of which were closed by sulfonylureas (Heissig et al. 2005; Urban and Panten 2005; Panten et al. 2013; Schulze et al. 2017), argues against these views. First, KIC does not generate glycolytic glycerol 3-phosphate (see Fig. 1). Second, KIC amplified without rise in the islet NADPH/NADP^+^ ratio (Panten and Rustenbeck 2008). The mitochondrial isocitrate dehydrogenase 2 apparently did not supply sufficient isocitrate to increase the cytosolic NADPH production via isocitrate dehydrogenase 1 (essential source of cytosolic NADPH in mouse islets, MacDonald 2002, see also Fig. 1). Moreover, there are also objections to amplification by mitochondrial export of malate, succinate, or alpha-ketoglutarate (Panten et al. 2013), but not to amplification by mitochondrial export of citrate and acetoacetate, which are both sources of cytosolic acetyl-CoA in insulin-secreting cells (Jitrapakdee et al., see also Fig. 1).

Assuming that the metabolic amplification is brought about by a final mechanism common to glucose and KIC, only mediation by cytosolic citrate and acetoacetate fits into this role. Therefore, we have expected that increases in fuel-induced amplification of insulin secretion coincide with increases in acetyl-CoA content. However, dissociations between fuel-induced amplification and increase in islet content of acetyl-CoA were observed after 20 min incubations (Panten et al. 2016). But these observations did not rule out that the supply of cytosolic acetyl-CoA was the essential mediator of metabolic amplification. This hypothesis was tested in the current study both at an early and later stage of stimulated secretion in normal mouse islets. The observations support the view that supply of cytosolic acetyl-CoA mediates metabolic amplification.

## Materials and methods

### Chemicals and media

Sigma/Fluka (Taufkirchen, Germany) provided N-ethylmaleimide, glutathione, dithiothreitol, oxaloacetate, acetylphosphate, phosphotransacetylase, citratesynthase, and acetyl-CoA (sodium salt). Sources of other chemicals and composition of basal medium were previously described (Panten et al. 2013).

### Islet isolation

Pancreatic islets were isolated from the pancreas of female albino mice (NMRI, 12–14 weeks old, fed an unrestricted diet) by injecting a collagenase solution (1.4 U per mL Krebs–Ringer medium) into the common bile duct and hand-picking the islets under a stereomicroscope after 10 min of digestion (for details, see Willenborg et al. 2012). The composition of the HEPES-buffered Krebs–Ringer medium was (mmol/L) NaCl (118.5), KCl (4.7), CaCl_2_ (2.5), KH_2_PO_4_ (1.2), MgSO_4_ (1.2), NaHCO_3_ (20), HEPES (10), and BSA 0.2% w/v. The glucose concentration was 5 mmol/L. The study was conducted in accordance with the Principles of Laboratory Care, approved by the responsible authority of the State of Lower Saxony, Germany.

### Insulin secretion

Batches of 50 freshly isolated islets were perifused in a purpose-made chamber thermostated at 37 °C. The flow rate was 0.9 mL/min; the perifusion medium was a HEPES-buffered Krebs–Ringer medium as described above, which was saturated with 95% O_2_ and 5% CO_2_ and contained the respective secretagogue. The insulin content of the fractionated efflux was determined by ELISA according to the manufacturer’s protocol (Mercodia, Uppsala, Sweden).

### Content of acetyl-CoA and acetyl-CoA plus CoA-SH

The islet content of acetyl-CoA and acetyl-CoA plus CoA-SH was measured by a modified version of an enzymatic cycling method, which produced citrate proportional to the original amount of acetyl-CoA and acetyl-CoA plus CoA-SH (Kato 1975). As described previously (Panten et al. 2013), groups of 15 size-matched freshly isolated islets were preincubated and incubated in basal medium without or with additional substances. Each single experiment of an experimental series consisted of simultaneous preincubation (60 min) of 2 or 3 batches of size-matched islets (15 islets per batch), followed by incubation (5 or 20 min). Incubations in 200 µL medium were stopped by centrifuging the incubation tube and removing 190 µL of medium (Panten et al. 2013). There was no washing step: to the 10 µL of medium containing the islets were added 40 µL of 15 mmol/L HCl, followed by vortex mixing, 5 min at 97 °C, cooling for 5 min (water of room temperature), and centrifuging for 5 min (20,000 g, 2 °C). The batches of a single experiment were analyzed simultaneously.

For measurement of acetyl-CoA, 20 µL of supernatant (or appropriate dilutions) plus 5 µL N-ethylmaleimide (0.1 mmol/L, dissolved in 0.5 mol/L Tris–HCl-buffer, pH 7.6) was vortexed, incubated for 12 min at 30 °C, and cooled (ice bath). Cycling followed without delay and was started by adding 25 µL cycling reagent (100 mmol/L Tris–HCl-buffer, pH 7.4, 40 mmol/L NH_4_^+^, 20 µmol/L glutathione, 2.4 mmol/L oxaloacetate, 4 mmol/L acetyl phosphate, 0.04% BSA, 64 U/mL phosphotransacetylase, 8 U/mL citrate synthase) and vortex mixing. Cycling at 30 °C lasted 60 min and was stopped by 5 min at 97 °C, followed by cooling (ice bath) and centrifuging for 5 min (20,000 g, 2 °C). The citrate in aliquots of the supernatant was measured as described (Panten et al. 2013) with minor modification.

For the measurement of acetyl-CoA plus CoA-SH, 20 µL of the acid 20,000 g supernatant (or appropriate dilutions) plus 5 µL dithiothreitol (20 mmol/L, dissolved in 0.5 mol/L Tris–HCl-buffer, pH 7.6) were vortexed, incubated for 12 min at 30 °C, and cooled on ice. Cycling (reagent contained no glutathione) and citrate measurement were carried out as described above.

Checking of the method was carried out by appropriate extra experiments. Controls were carried out by incubation in preincubation medium. The amounts of acetyl-CoA and acetyl-CoA + CoASH in the 2 or 3 batches of a single experiment were reflected in the amounts of citrate, which were produced by cycling and were given as normalized content of acetyl-CoA or acetyl-CoA plus CoA-SH (control = 100%). When giving the acetyl-CoA content as fmol/islet, appropriate standards were applied, starting at the step of HCl treatment.

### Statistical analysis

Values are presented as mean ± SEM. Means were compared by Wilcoxon’s matched-pairs signed rank test or Friedman’s test, followed by appropriate post hoc tests, including the Bonferroni-Holm procedure for multiple comparisons. When noted, the *U*-test of Wilcoxon and of Mann and Whitney was used. All tests were two-tailed and significance was assumed at *P* < 0.05.

## Results

### Necessity of immediate measurement of the acetyl-CoA content

The present study includes measurements of the islet content of acetyl-CoA and of acetyl-CoA plus CoA-SH after 5 min incubations to assess the relevance for the first phase of stimulated secretion. To enable meaningful 5-min tests, the previously performed washing step, which took ~ 2 min after finishing the incubation, was omitted. Measured this way, the acetyl-CoA content of islets after 20 min of incubation in the absence of exogenous fuels was 36.5 ± 3.5 fmol/islet (*n* = 8) which is virtually double the content found earlier after the same incubation when a washing step was included (18.2 ± 1.7 fmol/islet, *n* = 6, Panten et al. 2016). This points to fast changes in acetyl-CoA metabolism and to removal of loose cells from the outer tissue layer of islets during the washing step.

### Acetyl-CoA contents after different preincubation conditions

The experimental protocol to abolish the metabolic amplification by glucose but not by KIC requires a preincubation for 60 min in the absence of exogenous fuel and the presence of a sulfonylurea to depolarize the beta cells (see above, Fig. 2). Therefore, acetyl-CoA contents were compared after 65 min incubations, including the 5 min incubation time of controls. The content after incubation in the absence of exogenous fuels was 38.4 ± 4.2 fmol/islet (*n* = 8). The content after incubation in the absence of exogenous fuel and concomitant presence of the sulfonylurea glipizide at a maximally effective concentration (2.7 µmol/L) was not significantly different, namely 40.3 ± 2.2 fmol/islet (*n* = 8). Finally, the consequences of incubation in the presence of 3 mmol/L glucose were characterized. At this concentration, glucose is non-stimulatory, since the triggering signal is lacking (Henquin 2000). Here, an increased content was found, 89.0 ± 14.2 fmol/islet (*n* = 8), significantly more (*P* < 0.01, Mann–Whitney *U*-test) than after the same incubation time in the absence of exogenous fuel.

### Effects of alpha-ketoisocaproate or glucose after preincubation in the absence of exogenous fuel and concomitant presence of glipizide

**Fig. 3:**
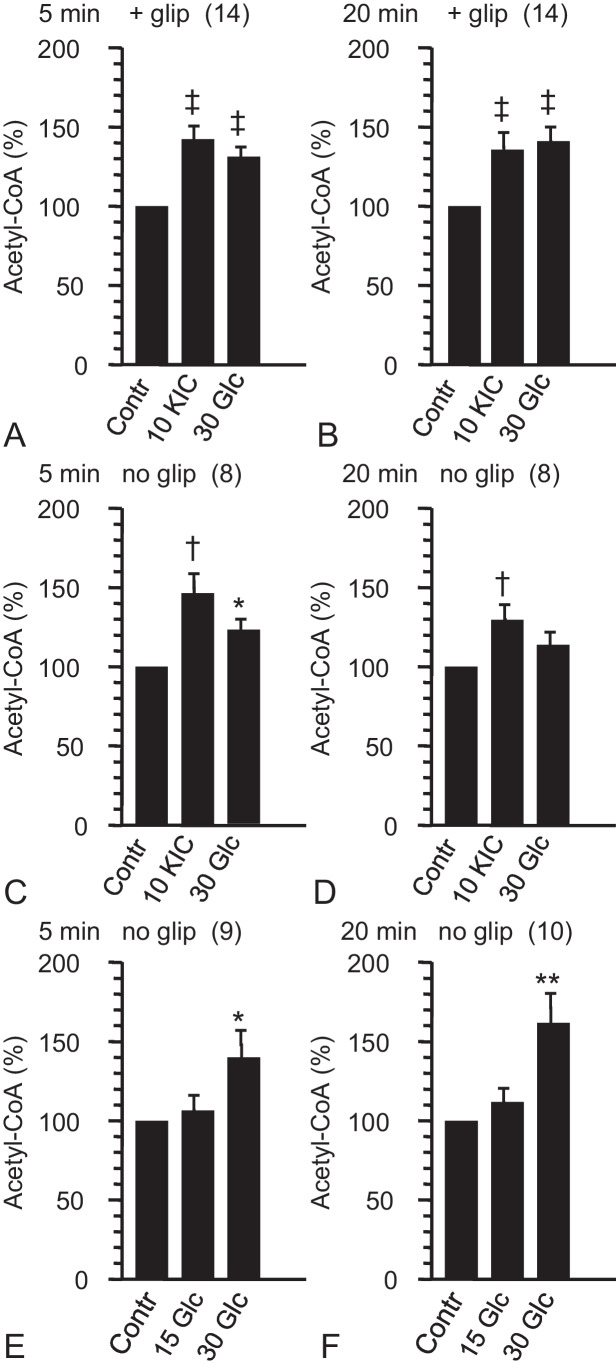
Effects of glucose (Glc) and alpha-ketoisocaproate (KIC) on the content of acetyl-CoA in mouse pancreatic islets. Each single experiment of an experimental series consisted of a parallel preincubation (60 min) of 3 batches of size-matched islets (15 islets per batch) in basal medium without substrate (**A**–**D**) or with 3 mmol/L Glc (**E**, **F**) followed by incubation (5 or 20 min as indicated at top) in preincubation medium (Contr, control) or in basal medium with substrate as indicated at bottom (10 KIC, basal medium with 10 mmol/L KIC; 15 or 30 Glc, basal medium with 15 or 30 mmol/L Glc). Glipizide (2.7 µmol/L) was absent (no glip) or present (+ glip) during the preincubations and incubations. In each single experiment, the contents were normalized (Contr = 100%). Data are mean ± SEM of results from *n*-independent experiments (*n* given in parentheses at top). *P < 0.05, **P < 0.02, †*P* < 0.01, and ‡*P* < 0.005 for comparison of the corresponding means of non-normalized values (substrate vs. control). The non-normalized values of citrate generated from acetyl-CoA by the cycling reaction are given in Table 1

**Fig. 4:**
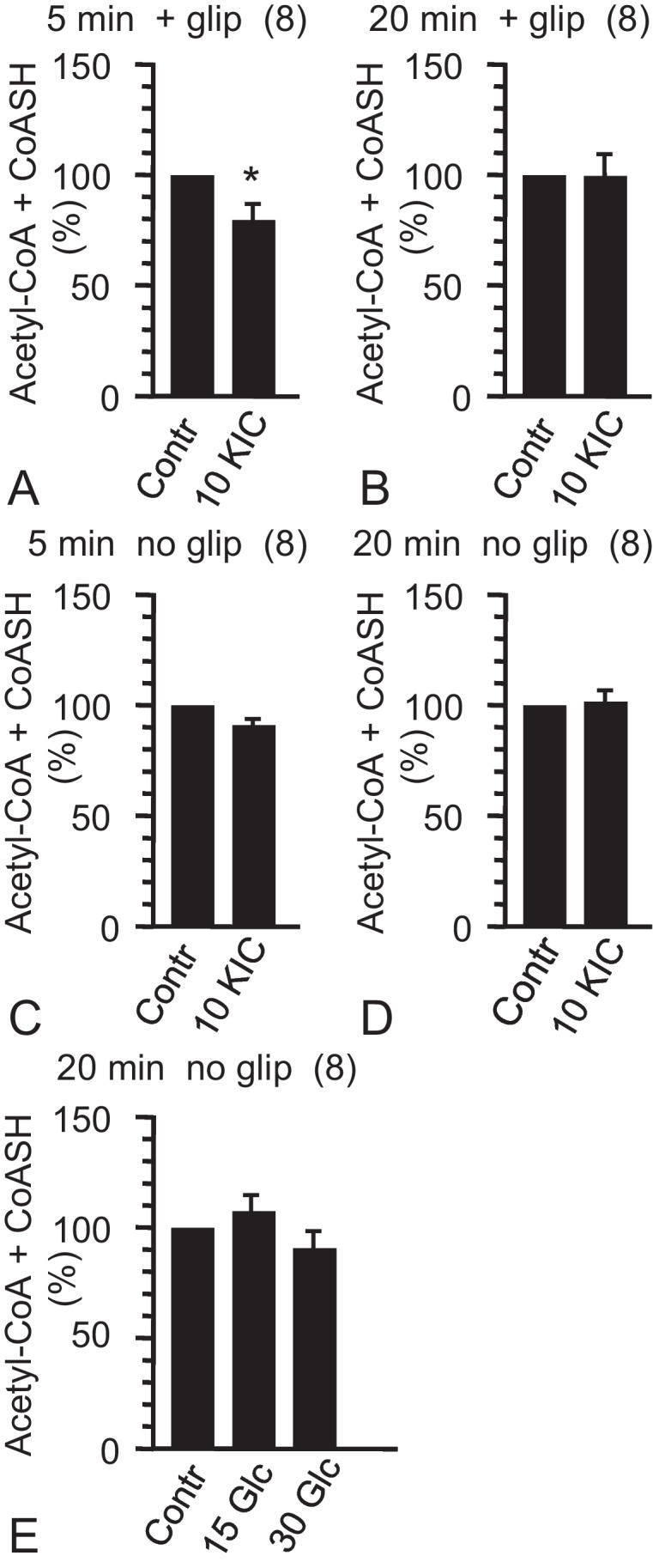
Effects of alpha-ketoisocaproate (KIC) and glucose (Glc) on the content of acetyl-CoA plus CoA-SH in mouse pancreatic islets. The preincubation medium contained no substrate (**A**–**D**) or 3 mmol/L Glc (**E**). In each single experiment of an experimental series (2 or 3 batches of 15 size-matched islets), the contents were normalized (Contr = 100%). Data are mean ± SEM of results from *n*-independent experiments (*n* given in parentheses at top, substrate in incubation media at bottom). *P < 0.05 for comparison of the corresponding means of non-normalized values (substrate vs. control). For further details and abbreviations, see legend of Fig. 3. The non-normalized values of citrate generated from acetyl-CoA plus CoA-SH by the cycling reaction are given in Table 2

In islets exposed to glipizide throughout the experiment and pretreated in the absence of exogenous fuel, KIC (10 mmol/L) increased the islet content of acetyl-CoA significantly within 5 min by about 40% as compared with the content after continued incubation in the preincubation medium (Fig. 3A, B). The relative increase vs. control was slightly diminished but still highly significant after 20 min incubation (Fig. 3A, B). Under the same conditions, KIC decreased the acetyl-CoA plus CoA-SH content vs. control within 5 min, but not after 20 min (Fig. 4A, B). When 30 mmol/L glucose was used instead of 10 mmol/L KIC, practically the same degree of acetyl-CoA increase as with KIC was observed, both after 5 and 20 min of incubation (Fig. 3A, B).

**Table 1:** Effects of alpha-ketoisocaproate (KIC) and glucose (Glc) on the citrate content (pmol/islet) produced by cycling of the islet content of acetyl-CoA (corresponds to Fig. 3)

	Fuel (mmol/L)	Fuel (mmol/L)	Fuel (mmol/L)
A 5min, + glip	Control (no fuel)	KIC (10 mmol/L)	Glc (30 mmol/L)
*n* = 14	115.3 ± 11.2	161.0 ± 16.5^d^	149.1 ± 7.4^d^
B 20 min, + glip	Control (no fuel)	KIC (10 mmol/L)	Glc (30 mmol/L)
*n* = 14	78.2 ± 8.5	104.3 ± 16.5^d^	107.8 ± 12.7^d^
C 5 min, no glip	Control (no fuel)	KIC (10 mmol/L)	Glc (30 mmol/L)
*n* = 8	140.6 ± 20.5	197.9 ± 25.3^c^	173.8 ± 29.8^a^
D 20 min, no glip	Control (no fuel)	KIC (10 mmol/L)	Glc (30 mmol/L)
*n* = 8	177.2 ± 17.2	210.4 ± 33.2^c^	197.0 ± 19.7^0^
E 5 min, no glip	Control (3 mmol/L Glc)	Glc (15 mmolL)	Glc (30 mmol/L)
*n* = 9	173.0 ± 16.5	175.7 ± 13.2^0^	240.2 ± 33.4^a^
F 20 min, no glip	Control (3 mmol/L Glc)	Glc (15 mmolL)	Glc (30 mmol/L)
*n* = 10	221.5 ± 31.2	244.1 ± 42.2^0^	342.8 ± 19.0^b^

Each single experiment of an experimental series consisted of simultaneous preincubation (60 min) of 3 batches of size-matched islets (15 islets per batch) in basal medium without substrate (A-D) or with 3 mmol/L Glc (E, F) followed by incubation (5 or 20 min as indicated on the left) in preincubation medium (Con, control) or in basal medium with substrate as indicated for each series. Glipizide (2.7 µmol/L) was absent (no glip) or present (+ glip) during the preincubations and incubations. Data are mean ± SEM of results from separate experiments (n given on the left). ^a^*P* < 0.05, ^b^*P* < 0.02, ^c^*P* < 0.01, ^d^*P* < 0.005 and ^0^*P* > 0.05 for comparison of the corresponding means (substrate vs control)

**Table 2:** Effects of alpha-ketoisocaproate (KIC) and glucose (Glc) on the citrate content (pmol/islet) produced by cycling of the islet content of acetyl-CoA + CoASH (corresponds to Fig. 4)

	Fuel (mmol/L)	Fuel (mmol/L)	Fuel (mmol/L)
A 5min, + glip	Control (no fuel)	KIC (10 mmol/L)	
*n* = 8	207.7 ± 14.4	160.0 ± 13.4^a^	
B 20 min, + glip	Control (no fuel)	KIC (10 mmol/L)	
*n* = 8	205.5 ± 14.1	195.1 ± 7.2	
C 5 min, no glip	Control (no fuel)	KIC (10 mmol/L)	
*n* = 8	261.2 ± 17.4	233.2 ± 9.7^o^	
D 20 min, no glip	Control (no fuel)	KIC (10 mmol/L)	
*n* = 8	211.5 ± 14.9	213.0 ± 19.5^o^	
E 20 min, no glip	Control (3 mmol/L Glc)	Glc (15 mmolL)	Glc (30 mmol/L)
*n* = 8	213.8 ± 15.8	224.1 ± 13.7^o^	187.2 ± 14.7^o^

Each single experiment of an experimental series consisted of simultaneous preincubation (60 min) of 3 batches of size-matched islets (15 islets per batch) in basal medium without substrate (A-D) or with 3 mmol/L Glc (E) followed by incubation (5 or 20 min as indicated on the left) in preincubation medium (Con, control) or in basal medium with substrate as indicated for each series. Glipizide (2.7 µmol/L) was absent (no glip) or present (+ glip) during the preincubations and incubations. Data are mean ± SEM of results from separate experiments (n given on the left). ^a^*P* < 0.05, and ^0^*P* > 0.05 for comparison of the corresponding means (substrate vs control)

### Effects of alpha-ketoisocaproate or glucose after preincubation in the absence of exogenous fuel

When 10 mmol/L KIC was added to islets which had been preincubated in the absence of exogenous fuel, the islet content of acetyl-CoA increased significantly within 5 min by nearly 50% as compared with the content after continued incubation in the preincubation medium (Fig. 3C, D). The relative increase vs. control was diminished but still significant after 20 min incubation (Fig. 3C, D). These increases in acetyl-CoA content by KIC were not accompanied by significant changes in the islet content of acetyl-CoA plus CoA-SH (Fig. 4C, D). When 30 mmol/L glucose was used instead of 10 mmol/L KIC, the islet content of acetyl-CoA increased significantly within 5 min by about 25% as compared with the continued incubation in the preincubation medium, but was no longer significantly elevated after 20 min of incubation (Fig. 3C, D).

### Concentration-dependent effects of glucose after preincubation in the presence of a non-stimulatory fuel concentration

Two glucose concentrations, 15 mmol/L and 30 mmol/L, were used to characterize the changes in the acetyl-CoA content after a 60 min preincubation in the presence of 3 mmol/L glucose, a non-stimulatory concentration. Raising the glucose concentration to 15 mmol/L did not significantly increase the acetyl-CoA content beyond the elevated level established by 3 mmol/L (see above). This was true for both, 5 min and 20 min incubations (Fig. 3E, F). Raising the glucose concentration to 30 mmol/L significantly increased the islet content of acetyl-CoA within 5 min by about 40%. The relative increase as compared with the content after continued incubation in the preincubation medium was even larger after 20 min and amounted to more than 60% (Fig. 3E, F). Neither 15 nor 30 mmol/L glucose affected the content of acetyl-CoA + CoA-SH after incubation for 20 min (Fig. 4E).

**Fig. 5:**
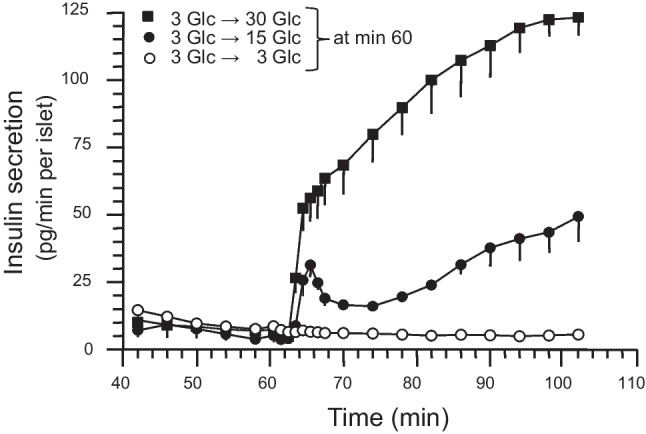
Effects of glucose (Glc) on the kinetics of insulin secretion by mouse pancreatic islets. During the control period (time 0 to 60 min), the islets were perifused with medium containing 3 mmol/L Glc (3 Glc) and during the test period (time 61 to 104 min) with medium containing 30 mmol/L Glc (30 Glc, black squares, *n* = 5), 15 mmol/L Glc (15 Glc, black circles, *n* = 4), or 3 mmol/L Glc as control (3 Glc, white circles, *n* = 5). Data points are the mean of *n*-independent experiments (with SEM shown when larger than symbols) and are plotted in the middle of the sampling intervals. In each single experiment, the secretion rates by 30 or 15 mmol/L glucose were continuously higher from 63.5 or 64.5 min, respectively, to 102 min than the corresponding prestimulatory rate at 58 min

Since the kinetics of insulin secretion under these experimental conditions were not known (in contrast to the experiments depicted in Fig. 2), it was characterized by perifusion. Raising the glucose concentration from 3 to 15 mmol/L led to a biphasic response, where the maximum of the first phase was reached after 6 min and a nadir occurred at 14 min, followed by a slowly ascending second phase thereafter (Fig. 5). Raising the glucose concentration from 3 to 30 mmol/L led to a response where the steep, first phase-like increase was followed by the ascending second phase without an intermittent minimum (Fig. 5).

## Discussion

### Methodological considerations

This study presents evidence that the KIC-induced amplification of insulin secretion coincides with the rapid and sustained increase in supply of cytosolic acetyl-CoA. This result was obtained by measuring the islet content of acetyl-CoA after 5 min and 20 min incubations with KIC and omitting the time-consuming washing step thereafter. Measured this way, the acetyl-CoA content of islets was practically twice as high as measured earlier under the same experimental conditions (Panten et al. 2013, 2016). Irrespective of the washing step, normalized (control = 100%) increases in the islet content of acetyl-CoA induced by KIC or glucose within 20 min were pronounced in the presence of glipizide (Panten et al. 2016, Fig. 2A as compared with current Fig. 3B), but moderate for KIC or insignificant for glucose in the absence of glipizide (Panten et al. 2016, Fig. 2E as compared with current Fig. 3D). So, a typical earlier result was reproducible in spite of the omission and, at the same time, meaningful measurements of early changes could be performed.

### Subcellular localization of acetyl-CoA

The metabolic fate of the alpha-keto acids in beta-cell mitochondria is largely known, facilitating conclusions as to the subcellular localization of the changes of the acetyl-CoA content reported here. In mouse islets exposed to glipizide throughout the experiment and pretreated without exogenous fuel, the efficacy of 10 mmol/L alpha-ketoisovalerate (KIV) to amplify the secretion of insulin after 20 min was much lower than the one of 10 mmol/L KIC (see Fig. 2). The following observations explain the differences between KIV and KIC. First, pyruvate dehydrogenase is inhibited by the catabolites of both alpha-ketoacid anions, as was observed in hepatocytes (Walajtjs-Rode and Williamson, 1980). Second, KIV generates acetyl-CoA only via pyruvate dehydrogenase, whereas KIC generates acetyl-CoA also via its strong oxidation (Lenzen and Panten 1980, see also Fig. 1). Therefore, the similar increases in the islet content of acetyl-CoA produced by KIV and KIC after 20 min (Panten et al. 2016) suggest that the mitochondrial acetyl-CoA content is of minor relevance for the observed changes. Furthermore, in mouse islets, mitochondria account only for ca. 4% of the beta-cell volume, whereas the cytosol accounts for ca. 50% (Dean 1973). So, for changes of the mitochondrial acetyl-CoA to significantly affect the measurements of islet contents, they have to be an order of magnitude higher than the cytosolic changes.

Acetyl-CoA is also located in the peroxisomes and the nucleus (Pehar and Puglielli 2013). The peroxisomes are unlikely to take up acetyl-CoA from the cytosol (Antonenkov and Hiltunen 2006) and the nucleus, which makes up to 12% of the mouse beta-cell volume (Dean 1973), forms a common compartment with the cytosol for acetyl-CoA. We previously assumed that increases in the islet acetyl-CoA content after incubation for 20 min reflected acetyl-CoA uptake into the Golgi/ER and not the cytosol (Panten et al. 2016). The following considerations argue against our earlier hypothesis. Uptake of acetyl-CoA into the Golgi/ER from the cytosol is driven by the concentration gradient between the cytosol and the Golgi/ER, the volume of which is in beta-cells about eightfold smaller than that of the cytosol (Dean 1973). If the increase in the total acetyl-CoA content after 20 min reflected acetyl-CoA taken up into the Golgi/ER, increase in consumption of cytosolic acetyl-CoA by production of metabolites (see Fig. 1) should have eliminated the KIC-induced rise in the cytosolic acetyl-CoA concentration nearly completely at min 5, since in islets exposed to glipizide KIC had not decreased the total acetyl-CoA content after 5 min (Fig. 3A, B). But the consumption of cytosolic acetyl-CoA starts later than the strong and continuous supply by KIC (10 mmol/L). Moreover, limited supply of cytosolic NADPH during amplification by KIC (see “Introduction”) is expected to slow down the acetyl-CoA consuming synthesis of non-acetyl-CoA thioesters. Therefore, the period of consumption was too short for sufficient elimination of acetyl-CoA within 5 min. Hence, the increases in acetyl-CoA content by KIC mainly reflected increases in the cytosolic acetyl-CoA.

In islets exposed to glipizide, 5 min exposure to 10 mmol/L KIC significantly decreased the acetyl-CoA plus CoA-SH content (Fig. 4A), suggesting that CoA-SH was consumed by increase in thioester production. CoA-SH can be trapped by a rapid rise in the cytosolic production of 3-hydroxy-3-methylglutaryl-CoA (HMG-CoA), malonyl-CoA, and fatty acyl-CoA, synthesized by fuel-induced supply of cytosolic acetyl-CoA (MacDonald et al. 2005, Prentki et al. 2013, see also Fig. 1). After 20 min exposure to KIC, the acetyl-CoA plus CoA-SH content was no longer decreased (Fig. 4B), presumably due to more rapid consumption of non-acetyl-CoA thioesters than at minute 5. The outcome of the acetyl-CoA plus CoA-SH measurements was not changed by the increases in the acetyl-CoA content (Fig. 3A, B), since these increases involved trapping of corresponding amounts of CoA-SH via citrate lyase and acetoacetyl-CoA synthetase (see Fig. 1). The evidence that KIC promoted non-acetyl-CoA thioester synthesis renders our earlier assumption unlikely that the increases in acetyl-CoA content reflected reduced turnover of acetyl-CoA pools (Panten et al. 2016). However, coincidence with increases in acetyl-CoA content (Fig. 3A, B) indicated that the effects on acetyl-CoA plus CoA-SH content resulted from KIC-induced increase in supply of cytosolic acetyl-CoA.

### Comparison of KIC with glucose: differences and similarities

In islets exposed to glipizide and preincubated without exogenous fuel, the pyruvate carboxylase was inhibited and glucose was unable to amplify the secretion (Panten et al. 2013). Under this condition however, where weak supply of cytosolic acetyl-CoA by glucose is expected, glucose elevated the acetyl-CoA content as intensively as KIC (Fig. 3A, B). This suggests that glucose enabled only slow and weak acetyl-CoA-consuming syntheses of non-acetyl-CoA thioesters.

In islets preincubated without exogenous fuel, the acetyl-CoA content was less elevated after 20 min than after 5 min, both with KIC and with glucose as stimuli (Fig. 3C, D). This may indicate that the supply of cytosolic acetyl-CoA rose more rapidly than the acetyl-CoA consumption by synthesis of non-acetyl-CoA thioesters and uptake into the Golgi/ER. This view is supported by the failure of KIC to cause a significant decrease in acetyl-CoA plus CoA-SH content under this condition (Fig. 4C, D).

In islets pretreated with 3 mmol/L glucose, raising the glucose concentration from 3 to 15 mmol/L failed to induce a significant elevation of the islet acetyl-CoA content (Fig. 3E, F), but coincided with considerable insulin release (Fig. 5). Presumably increase in synthesis of non-acetyl-CoA thioesters and uptake into the Golgi/ER nearly completely consumed the cytosolic acetyl-CoA supplied by glucose. The increase in acetyl-CoA content by raising the glucose concentration from 3 to 30 mmol/L (Fig. 3E, F) may then reflect a more rapidly increasing supply than consumption of cytosolic acetyl-CoA.

The increase in acetyl-CoA content by 30 mmol/L glucose differs from the observation in an earlier study, where the acetyl-CoA content of perifused rat islets pretreated for 30 min with 2.5 mmol/L glucose was not increased after 3 min with 25 mmol/L glucose and was even decreased after 30 min (Liang and Matschinsky 1991). Also, the first-phase insulin secretion in this study was more pronounced than that induced by 30 mmol/L glucose in the present study (see Fig. 5). A straightforward explanation for these phenomena would be that the rate of cytosolic acetyl-CoA consumption by synthesis of non-acetyl-CoA thioesters was higher in rat islets than in mouse islets. The inability of glucose stimulation to change the islet content of acetyl-CoA plus CoA-SH (Fig. 4E) and of CoA-SH (Liang and Matschinsky 1991) does not rule out trapping of CoA-SH, since CoA-SH can be generated from acyl-CoA during glucose-stimulated triacylglycerol synthesis on the ER (Prentki et al. 2013; Lorenz et al. 2013).

Collectively, the previous and the present findings indicate that the islet content of acetyl-CoA did not always correlate with the glucose-induced amplification of insulin secretion, but the findings are consistent with amplification by increased supply of cytosolic acetyl-CoA.

As strengthened by the present findings, the supply of cytosolic acetyl-CoA is well suited to provide signals, which amplify the insulin secretion. The following considerations narrow down these signals: (1) The failure of 15 mmol/L glucose to induce significant increase in the islet content of acetyl-CoA (Fig. 3E, F) despite stimulation of insulin release (Fig. 5) argues against amplification by direct action of acetyl-CoA (without molecular transformation). (2) Protein isoprenylation and cholesterol production are not acute regulatory events (Metz et al., 1993, Zúñiga-Hertz et al. 2015) and stimulation of malonyl-CoA and fatty acyl-CoA synthesis is not always required for amplification (Chakravarthy et al. 2007; Cantley et al. 2019). (3) The numerous lysine-acetylated proteins in the islet cytosol (Zhang et al. 2019) indicate that acetyl-CoA serves as substrate for cytosolic protein acetylation. The rate of acetylation appears to be determined by the concentration of acetyl-CoA relative to the concentration of CoA-SH in the immediate vicinity of the lysine acetyltransferases, since CoA-SH is known to exert a product inhibition on most of the lysine acetyltransferases (Albaugh et al. 2011; Chaudhary et al. 2014; Drazic et al. 2016). 4. The wash-out of fuel secretagogues at stimulatory concentrations abolished the amplification of insulin secretion within 14 min (Panten et al. 2016). This reversibility of the metabolic amplification fits to the rapidly reversible protein acetylation by the action of lysine deacetylases.

In conclusion, the present observations indicate that glucose as well as KIC increase the supply of acetyl-CoA in the beta-cell cytosol during both phases of insulin secretion. This situation enables intensified protein acetylation, whereby the supply of cytosolic acetyl-CoA may function as a critical signal in the pathway of metabolic amplification. Identification of those cytosolic proteins which become acetylated during stimulated insulin secretion will give insight into the mechanisms which specifically promote the amplification of secretion and are not merely permissive or supportive. Clarification of these issues is not only relevant for the physiology of beta-cell function but also of major importance for the pathophysiology of type 2 diabetes (Grespan et al. 2018).

## Data Availability

The data sets generated during and/or analyzed during the current study are not publicly availably but are available from the corresponding authors on reasonable request.
